# Assessing Patient Readiness for an Electronic Patient-Reported Outcome–Based Symptom Management Intervention in a Multisite Study

**DOI:** 10.1200/OP.23.00339

**Published:** 2023-11-27

**Authors:** Roshan Paudel, Angela C. Tramontano, Christine Cronin, Sandra L. Wong, Don S. Dizon, Hannah H. Jenkins, Jessica Bian, Raymond U. Osarogiagbon, Deborah Schrag, Michael J. Hassett

**Affiliations:** ^1^ Dana-Farber Cancer Institute, Boston, MA; ^2^ Dartmouth-Hitchcock Medical Center, Lebanon, NH; ^3^ Lifespan Cancer Institute and Brown University, Providence, RI; ^4^ West Virginia University Cancer Center, Morgantown, WV; ^5^ Maine Medical Center, Portland, ME; ^6^ Baptist Memorial Healthcare Corporation, Memphis, TN; ^7^ Memorial Sloan Kettering Cancer Center, New York, NY

## Abstract

**PURPOSE:**

While the use of electronic patient-reported outcomes (ePROs) in routine clinical practice is increasing, barriers to patient engagement limit adoption. Studies have focused on technology access as a key barrier, yet other characteristics may also confound readiness to use ePROs including patients' confidence in using technology and confidence in asking clinicians questions.

**METHODS:**

To assess readiness to use ePROs, adult patients from six US-based health systems who started a new oncology treatment or underwent a cancer-directed surgery were invited to complete a survey that assessed access to and confidence in the use of technology, ease of asking clinicians questions about health, and symptom management self-efficacy. Multivariable ordinal logistic regression models were fit to assess the association between technology confidence, ease of asking questions, and symptom management self-efficacy.

**RESULTS:**

We contacted 3,212 individuals, and 1,043 (33%) responded. The median age was 63 years, 68% were female, and 75% reported having access to patient portals. Over 80% had two or more electronic devices. Most patients reported high technology confidence, higher ease of asking clinicians questions, and high symptom management self-efficacy (n = 692; 66%). Patients with high technology confidence also reported higher ease of asking nurses about their health (adjusted odds ratio [AOR], 4.58 [95% CI, 2.36 to 8.87]; *P* ≤ .001). Those who reported higher ease of asking nurses questions were more likely to report higher confidence in managing symptoms (AOR, 30.54 [95% CI, 12.91 to 72.30]; *P* ≤ .001).

**CONCLUSION:**

Patient readiness to use ePROs likely depends on multiple factors, including technology and communication confidence, and symptom management self-efficacy. Future studies should assess interventions to address these factors.

## Teaser

A study assessing patients' readiness to use ePRO in routine practice found a subset of patients with low confidence in using technology, asking questions, and low symptom management self-efficacy, suggesting access to technology alone may be inadequate to ensure all patients are ready to use an ePRO

## INTRODUCTION

In research settings, patient-reported outcomes (PROs) improve health-related quality of life,^1^ increase survival,^2^ improve patient-clinician communication,^3^ and improve patient well-being.^4,5^ There is a growing interest in systematically deploying electronic patient-reported outcomes (ePROs) embedded within electronic health records (EHRs) in routine oncologic care. Recognizing the need to assess the systematic implementation of ePROs in routine oncologic care, the Symptom Management of Patient-Reported Outcomes in Oncology (SIMPRO) Consortium was established with funding from the National Cancer Institute Cancer Moonshot Initiative. The goals of SIMPRO are to develop and integrate an ePRO system into the EHRs and workflows of the participating centers and determine the effectiveness of the program on health care utilization through a multisite clinical trial (ClinicalTrials.gov identifier: NCT03850912).^6^ To achieve these goals, a symptom management system Electronic Symptom Management Program (eSyM) was developed and integrated into the Epic EHR at six participating SIMPRO sites. eSyM enables the routine collection of patient-reported symptoms after surgery or chemotherapy from patients with a suspected or confirmed thoracic, gynecologic, or GI malignancy.^7^ eSyM was deployed via a modified stepped wedge protocol from September 2019 to March 2023. As of April 2023, more than 10,000 medical oncology and surgery patients have completed more than 90,000 symptom questionnaires via the eSyM program.

**CONTEXT**

**Key Objective**
To assess factors associated with patients' readiness to use an electronic health record (EHR)–integrated patient-reported outcome program in routine oncology practice.
**Knowledge Generated**
We found high levels of access to and confidence in using technology, confidence in asking nurses and doctors questions, and confidence in managing symptoms. However, a subset of patients reported low confidence in using technology, low confidence in asking questions, and low symptom management self-efficacy.
**Relevance**
These findings suggest that access to technology alone may be inadequate to ensure that patients are ready to use an EHR-integrated patient-reported outcome system. Future studies should test interventions tailored to patients who report low communication confidence and low symptom management self-efficacy.

Despite ongoing investments in health information technology, steady increases in patient portal use,^8,9^ and growing evidence supporting the benefits of ePROs, the systematic collection of ePROs in routine oncologic practice has been lackluster.^10-12^ The slow pace of ePRO adoption in routine clinical practice could be explained by several factors, including implementation complexities,^13^ limitations of the platforms used to collect ePROs,^14,15^ perceived disruptions to clinical workflow,^16^ limited resources at health systems to deploy ePROs,^15,17,18^ and, perhaps most importantly, barriers to patients' use of ePROs.

Efforts to assess patient readiness to use digital health tools, such as ePROs, have focused on access to the technology and devices needed for engagement, such as smartphones and internet service.^19^ Studies have suggested that many patients with cancer lack the time or may not feel well enough to engage with digital health tools.^20-22^ Other investigators have focused on characterizing acceptability, feasibility, and willingness to complete PRO questionnaires.^23,24^ However, few studies have simultaneously explored the technological, communication, and symptom management self-efficacy–related barriers in a large, multi-institutional cohort of patients with cancer. Symptom management self-efficacy is the patient's perceived ability to implement behaviors to prevent, recognize, and relieve symptoms.^25^ Reasoning that successful implementation of a patient-focused ePRO platform would require satisfying multiple components of patient readiness, we sought to assess patients' access to and confidence in the use of technology, patients' confidence in communicating with clinicians to ask questions, and patients' confidence with symptom management self-efficacy in the context of the SIMPRO consortium of six academic and community-based cancer centers.

## METHODS

### Self-Efficacy, Attainment of Information Needs, Symptom Burden, and Satisfaction With Care Survey

The Self-efficacy, Attainment of information needs, Symptom burden, and Satisfaction with care (SASS) surveys were developed as part of the SIMPRO project to assess characteristics associated with patients' readiness to use a fully EHR-embedded ePRO-based symptom management tool. The SASS surveys include items from multiple validated instruments including the Patient-Reported Outcomes Measurement Information System (PROMIS),^26^ Consumer Assessment of Healthcare Providers and Systems (CAHPS),^27^ Communication and Attitudinal Self-Efficacy (CASE-Cancer),^28^ and study-developed items. Recognizing the limitations of EHRs to collect information on patients' use of technology and symptom management self-efficacy, the SASS surveys were administered to a subset of SIMPRO-eligible patients outside the EHR, using REDCap. One of the primary goals of the SASS survey was to simultaneously explore the complex relationship between technological and perceptual barriers. In that context, the questions used were designed to help understand the relationship between (1) access to and (2) confidence in the use of technology, (3) self-reported confidence in patients' ability to work with clinicians to manage their symptom, and (4) symptom management self-efficacy. At all six SIMPRO sites, a subset of SIMPRO-eligible patients were invited to participate in the SASS surveys, which were administered as a one-time, 20-minute instrument. The SASS surveys were collected at two time points: before eSyM (pre-eSyM) was deployed, and after eSyM (post-eSyM) was deployed. While the pre-eSyM and post-eSyM SASS surveys shared many common items, there were several differences including readiness factors. This analysis focuses on the readiness factors of patients who completed the pre-eSyM SASS surveys.

### Patients

The eSyM program was designed to address the symptoms of two patient populations: (1) patients with a GI, thoracic, or gynecologic malignancy who recently started a new chemotherapy treatment plan and (2) patients who were recently discharged after having surgery for a suspected GI, thoracic, or gynecologic malignancy. Case identification occurred automatically using two Epic-based, eSyM-created patient registries. Since eSyM was deployed as part of routine care, consent to participate in eSyM was not required. After the registries were activated and before eSyM was rolled out to patients, a randomly selected subset of eSyM-eligible patients were invited to complete the pre-eSyM SASS survey. A waiver of documentation of informed consent was obtained, and all patients participating in the SASS survey received an informational consent letter.

### Covariates and Outcomes

Demographic and clinical characteristics of respondents including their age, sex, race (White, Black, other race), ethnicity (Hispanic, non-Hispanic, unknown), marital status, cancer type, treatment modality, and Epic MyChart (patient portal) access were ascertained using data extracted from the EHR. Patient's educational attainment and employment status were gathered from the SASS survey.

Access to and confidence in using personal electronic devices were key independent covariates. Respondents were asked, *Please tell me if you have any of the following items: A tablet computer (with common brand names as examples), a desktop or laptop computer, a smartphone (with common brand names as examples)*. Response options were *yes*, *no*, and *don't know*. Personal electronic devices were quantified as one, two, or three devices, irrespective of the quantity in each item category. Similarly, respondents were asked *Overall, how confident do you feel using computers, smartphones, or other electronic devices to do the things you need to do online?* Four-level response options were *very confident*, *somewhat*, *only a little*, and *not at all confident*. For cross-tabular analysis, responders reporting *not at all* or *only a little* confident were classified as having low technology confidence; the rest were classified as having high confidence. Ease of asking clinicians questions was assessed using two items from the CASE-Cancer survey: (1) *It is easy for me to ask my doctors questions* and (2) *It is easy for me to ask nurses questions*. The four-level Likert response options were *strongly disagree*, *slightly disagree*, *slightly agree*, and *strongly agree*. For cross-tabular analysis, respondents reporting *strongly disagree* or *slightly disagree* were classified as having low confidence; the rest were classified as having high confidence in asking nurses questions. Self-efficacy regarding one's ability to work with the care team to manage one's symptoms was assessed using the PROMIS self-efficacy question, *I can work with my doctor to manage my symptoms*, with five response options: *not at all*, *a little*, *somewhat*, *quite*, and *very* confident. Respondents reporting *not at all*, *a little*, or *somewhat* were classified as having low symptom management self-efficacy; the rest were classified as having high symptom management self-efficacy.

### Statistical Analysis

Counts and proportions were assessed for dichotomous and polychotomous variables. Median and IQR were assessed for continuous variables. This study aimed to characterize patient readiness for ePROs. To assess readiness, we explored the extent to which technology access and technology confidence were associated with greater odds of ease of asking clinicians questions and whether ease of asking questions was associated with greater odds of symptom management self-efficacy. To control for potential confounding, multivariable ordinal logistic regression models were used with an assumption of proportional odds. All models included age at diagnosis, sex, race, ethnicity, marital status, education, employment, cancer type, and SIMPRO site as covariates. We defined statistical significance as *P* values < .05 in a two-sided test. We conducted all statistical analyses using SAS version 9.4 (SAS Institute, Inc, Cary, NC).

## RESULTS

### Descriptive Statistics

Among the 3,212 individuals who were identified and contacted from six participating cancer centers, 1,043 (33%) completed the survey. The median age was 63 years (IQR, 18). Among respondents, 67% were female and 90% identified as White and 97% as non-Hispanic ethnicity. Fifty-three percent (n = 553) of respondents had undergone a surgical procedure; the rest had received chemotherapy. A plurality (n = 296, 28%) of respondents were diagnosed with GI cancer, followed by thoracic (n = 205, 20%) and gynecologic (n = 204, 20%) cancers. There were 230 (22%) respondents who had surgery but were determined to not have cancer postoperatively. Most, 75% (n = 786), reported having Epic MyChart access. Nineteen percent (n = 201) reported having at least one device, and over half (51%, n = 534) reported having at least three personal electronic devices. Forty-nine percent (n = 511) reported being very confident using technology, with only 16% reported having little or no confidence in using technology. Over 75% of respondents strongly agreed that *it is easy for me to ask my doctor questions* and *it is easy for me to ask nurses question*. However, only 43% expressed being very confident working with doctors to manage symptoms (Table 1). Cross-tabular analysis found that most patients reported high confidence in using technology, high level of ease of asking nurses questions, and high symptom management self-efficacy (n = 692, 66%). Furthermore, most patients (n = 966, 92%) reported the high level of ease of asking nurses questions; however, most patients who reported having a low level of ease of asking nurses questions (n = 55, 6%) also reported low symptom management self-efficacy (35 of 55, 64%; Table 2).

**TABLE 1.**
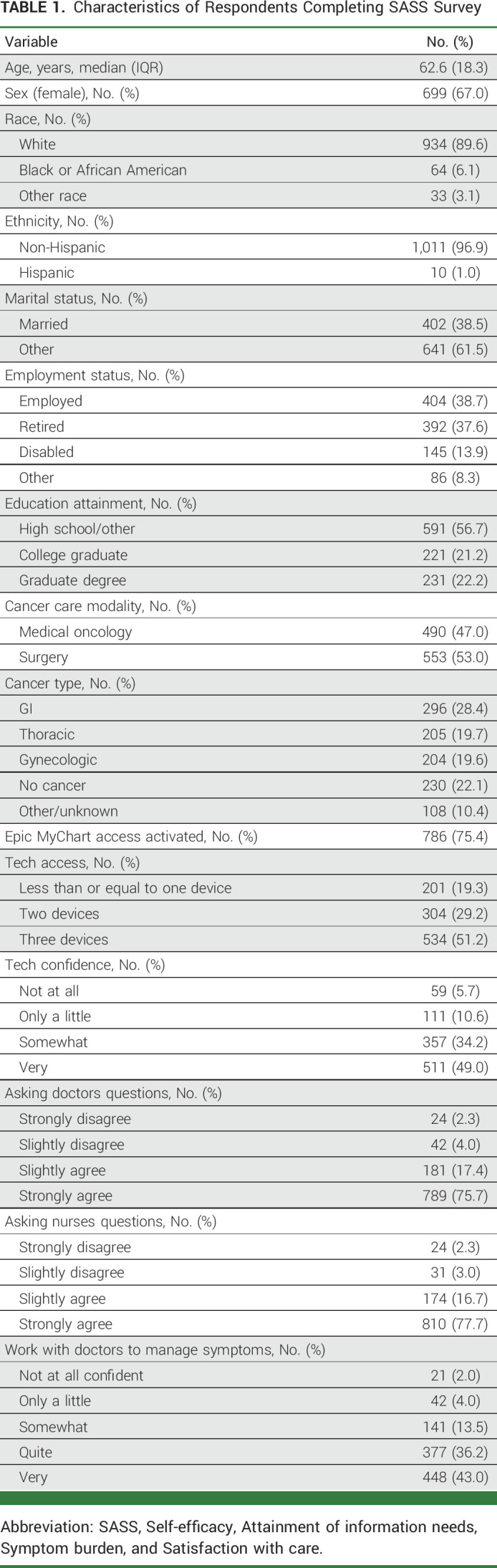
Characteristics of Respondents Completing SASS Survey

**TABLE 2.**
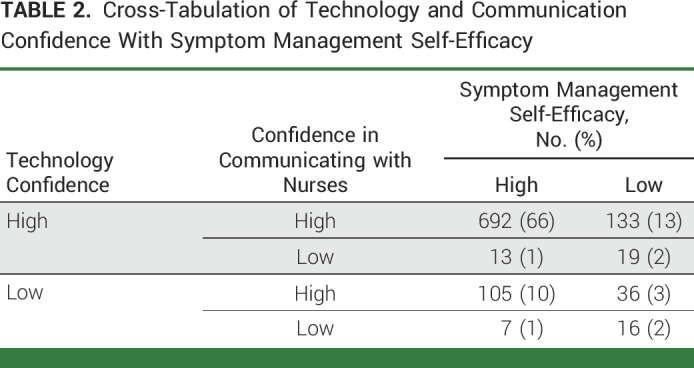
Cross-Tabulation of Technology and Communication Confidence With Symptom Management Self-Efficacy

### Multivariable Analyses

After adjusting for other covariates, having access to three or more electronic devices was not associated with a higher ease of asking doctors (adjusted odds ratio [AOR], 1.06 [95% CI, 0.45 to 2.51]; *P* = .90) or nurses questions (AOR, 1.40 [95% CI, 0.66 to 3.78]; *P* = .31; Table 3). However, being very confident in the use of personal electronic devices was associated with a higher ease of asking doctors (AOR, 2.80 [95% CI, 1.44 to 5.46]; *P* = .007) and nurses questions (AOR, 4.58 [95% CI, 2.36 to 8.87]; *P* ≤ .001; Table 3).

**TABLE 3.**
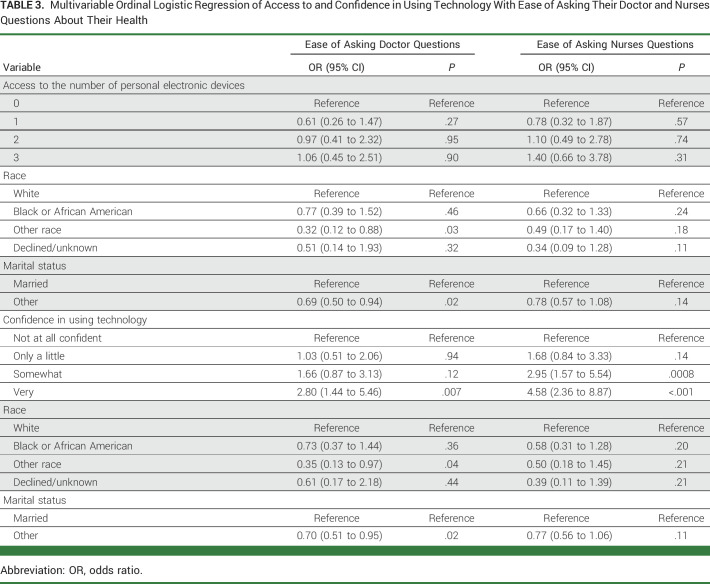
Multivariable Ordinal Logistic Regression of Access to and Confidence in Using Technology With Ease of Asking Their Doctor and Nurses Questions About Their Health

We assessed the association between the ease of asking doctors and nurses health-related questions with confidence regarding symptom management self-efficacy. Respondents who strongly agreed with the statement *it is easy for me to ask nurses questions* were 30-fold (AOR, 30.54 [95% CI, 12.91 to 72.30]; *P* < .001) more likely to report confidence in their ability to work with their doctors to manage their symptoms (Table 4). Similarly, respondents who strongly agreed with the statement *it is easy for me to ask my doctor questions* were 22-fold (AOR, 21.73 [95% CI, 9.40 to 50.26]; *P* ≤ .001) more likely to report confidence in their ability to work with their doctors to manage their symptoms (Appendix Table A1, online only). There were no statistically significant differences in the scores among the six SIMPRO sites (Appendix Table A2). In addition, there were no statistically significant differences in scores of medical oncology and surgery patients (Appendix Table A3).

**TABLE 4.**
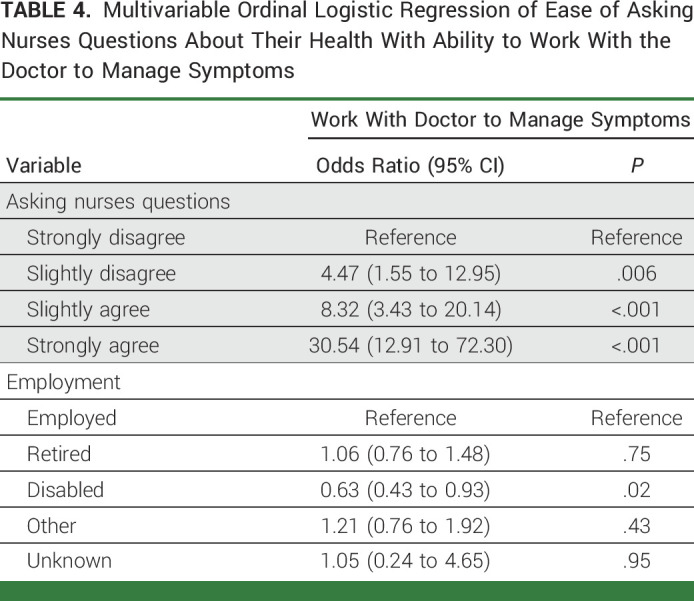
Multivariable Ordinal Logistic Regression of Ease of Asking Nurses Questions About Their Health With Ability to Work With the Doctor to Manage Symptoms

## DISCUSSION

In this study of an ePRO-naïve oncology patient population, higher levels of confidence in the use of technology and higher levels of ease of asking clinicians questions were associated with increased symptom management self-efficacy. We assessed patients' confidence with domains that may be important for patients' readiness to use ePROs in routine oncologic practice. Most patients reported high confidence across all three domains: (1) having confidence in the use of those devices, (2) having confidence in asking doctors and nurses questions about health, and (3) having confidence in working with doctors to manage symptoms. These characteristics suggest that a majority of our respondents expressed readiness to engage with a patient-directed symptom management program, such as eSyM. Despite these findings, we identified a subset of patients who reported low confidence in using technology and low symptom management self-efficacy. These traits might impair readiness to use ePROs. Another subset of patients reported low confidence in using technology and low ease of asking nurses questions. While this group was small, these patients also tended to report low symptom management self-efficacy, suggesting that they might face especially high barriers to engagement with a patient-directed, ePRO-based symptom management program, such as eSyM.

Previous studies have suggested that limited access to technology could serve as a barrier to patients' use of digital health technologies.^12,19,29^ We found that a relatively few patients (3.6%) lacked access to personal electronic devices and low access to devices was not a significant predictor of patients' self-reported ease of asking their doctors and nurses questions about their health. This finding suggests that technology and confidence barriers affect separate groups of patients. Self-reported confidence in using technology was a strong predictor of patients' ease of communicating with their providers reinforcing the delineation between access and confidence barriers. Patients who were *very confident* in their use of personal electronic devices were twice more likely to ask their providers for information about their health than those who were *not at all confident*.

Self-reported ease of asking questions were strong predictors of symptom management self-efficacy. In fact, patients who reported higher ease of asking questions were also highly likely to work with their doctors to manage their symptoms. This is an important finding as patients' self-reported ease, a sign of confidence, in asking questions may lead to increased levels of self-efficacy managing symptoms. Confidence is a marker of self-efficacy, a concept rooted in social cognitive theory.^30^ Self-efficacy is defined as an individual's perceived ability to perform a task or a specific behavior(s) to recognize and relieve symptoms^25^ and improve their health.^28^ Perceived ease of asking questions is an important aspect of self-efficacy, and self-efficacy is important to managing symptoms. However, it is noteworthy that more patients reported low symptom management self-efficacy and high confidence with communication, versus low symptom management self-efficacy and low confidence with communication, suggesting that efforts to improve communication confidence alone may be insufficient. This has an important implication, considering that previous studies documented an association between greater symptom management self-efficacy and higher quality of life.^31,32^ Lower levels of self-efficacy were associated with higher levels of pain, fatigue, anxiety, and poor physical functioning, among other bothersome symptoms.^33,34^ Patients with low self-efficacy may not feel empowered to communicate about their symptoms^35^ or may be unwilling to initiate conversations with their providers.^28^ Considering that patients who report low confidence with clinician communication and symptom self-management appear more likely to experience bothersome symptoms, an important goal of the SIMPRO project will be to determine if the eSyM program helps to overcome or exacerbates these barriers. Addressing the technology access barrier may make it easier for patients to use ePRO-based solutions, but low levels of confidence with communication and symptom management self-efficacy could undermine the successful implementation of this intervention. Future studies should develop and test interventions to help patients build self-confidence and improve symptom management self-efficacy.

There are several limitations to this study. First, multiple waves of the COVID pandemic affected hospital operations at different times across sites, making it challenging to control for changes in how surveys were administered. As such, temporal changes in patient population and COVID-related changes in practice patterns might have introduced unmeasured confounders. Second, only 33% of eligible patients responded to the survey. This is a major limitation, and thus, we cannot rule out nonresponse bias. For example, most sites administered SASS-control surveys electronically with no in-person option. This was done because of COVID-related restrictions. However, this might have decreased participation among those with limited access to a personal electronic device or the internet. This may be a more salient issue in rural and remote areas lacking broadband or robust Wi-Fi connectivity required to access the internet without disruption. Third, 90% of our respondents self-identified as White, and thus, our results may not be generalizable to other populations as the non-White population may face additional barriers. However, a majority of our respondents were high school graduates, indicating that our population may be more representative from a socioeconomic perspective. Finally, we did not stratify our sample on the basis of different age categories. It is plausible that older patients in our sample might have faced more barriers to respond to electronic surveys. Studies have shown that older adults are less likely to use digital technologies for health-related purposes.^36^

Despite these limitations, the present study broadens our understanding of the issues related to patients' readiness to use ePRO-based systems. Access to technology, as well as confidence in the use of technology, information seeking, and symptom management self-efficacy are all important markers of patient readiness to engage with ePRO-based symptom management programs. To date, few studies have examined these barriers in the context of patient readiness for ePROs. Moving forward, studies that further investigate how to help patients navigate these barriers might further advance the science around active symptom management using an ePRO platform in routine oncology practice.

In conclusion, the present study adds to the literature that access to technology alone is inadequate to successfully implement an ePRO system. Addressing other patient-level barriers will be critical for the successful implementation of an ePRO-based symptom management system. Patient-reported confidence and self-efficacy should be considered in the context of developing and deploying patient-directed digital health tools.

## Figures and Tables

**TABLE A1.**
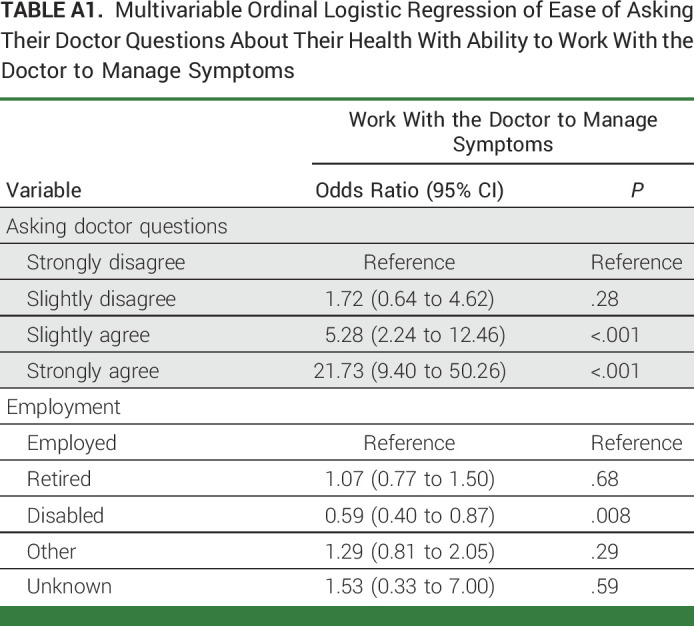
Multivariable Ordinal Logistic Regression of Ease of Asking Their Doctor Questions About Their Health With Ability to Work With the Doctor to Manage Symptoms

**TABLE A2.**
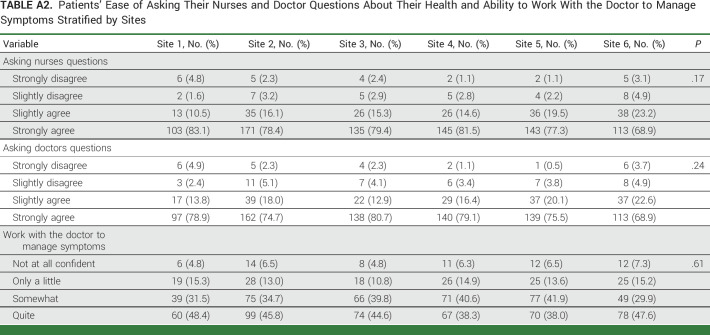
Patients' Ease of Asking Their Nurses and Doctor Questions About Their Health and Ability to Work With the Doctor to Manage Symptoms Stratified by Sites

**TABLE A3.**
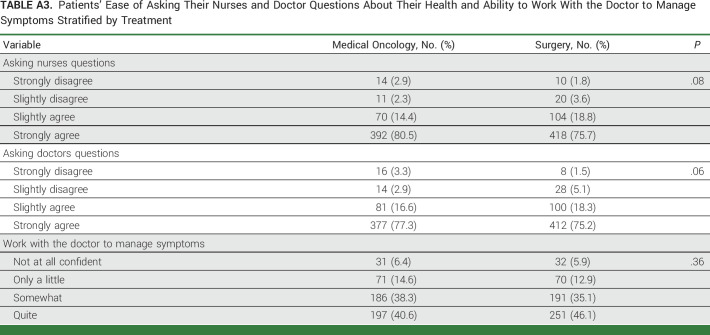
Patients' Ease of Asking Their Nurses and Doctor Questions About Their Health and Ability to Work With the Doctor to Manage Symptoms Stratified by Treatment
